# Common cortical responses evoked by appearance, disappearance and change of the human face

**DOI:** 10.1186/1471-2202-10-38

**Published:** 2009-04-24

**Authors:** Emi Tanaka, Koji Inui, Tetsuo Kida, Ryusuke Kakigi

**Affiliations:** 1 Department of Integrative Physiology, National Institute for Physiological Sciences, Myodaiji, Okazaki 444-8585, Japan; 2 Department of Physiological Sciences, School of Life Sciences, The Graduate University for Advanced Studies, Hayama, Kanagawa, Japan; 3 RISTEX, Japan Science and Technology Agency, Tokyo, Japan

## Abstract

**Background:**

To segregate luminance-related, face-related and non-specific components involved in spatio-temporal dynamics of cortical activations to a face stimulus, we recorded cortical responses to face appearance (Onset), disappearance (Offset), and change (Change) using magnetoencephalography.

**Results:**

Activity in and around the primary visual cortex (V1/V2) showed luminance-dependent behavior. Any of the three events evoked activity in the middle occipital gyrus (MOG) at 150 ms and temporo-parietal junction (TPJ) at 250 ms after the onset of each event. Onset and Change activated the fusiform gyrus (FG), while Offset did not. This FG activation showed a triphasic waveform, consistent with results of intracranial recordings in humans.

**Conclusion:**

Analysis employed in this study successfully segregated four different elements involved in the spatio-temporal dynamics of cortical activations in response to a face stimulus. The results show the responses of MOG and TPJ to be associated with non-specific processes, such as the detection of abrupt changes or exogenous attention. Activity in FG corresponds to a face-specific response recorded by intracranial studies, and that in V1/V2 is related to a change in luminance.

## Background

It has been proposed that there are specific neural processes underlying face perception. Functional magnetic resonance imaging (fMRI) and positron-emission tomography (PET) studies have shown that regions of the ventral occipito-temporal pathway of the brain, such as part of the fusiform gyrus (FG), called the fusiform face area (FFA), respond more to faces than other stimuli [1-8]. Intracranial electrophysiological recordings from the surface of the cortex have demonstrated a face-specific negative component maximum around 200 ms, N200, which was generated in the lateral part of the FG and at the border of the middle temporal gyrus and middle occipital gyrus in human patients [9-13]. Magnetoencephalography (MEG) studies have reported M100 evoked during 80–150 ms [14-16] and M200 or M170 evoked during 140–200 ms [14-22], which respond maximally to face stimuli. Numerous event-related potential (ERP) studies have also reported a negative component peaking 150–170 ms post-stimulus over temporo-parietal regions of the human scalp which responds maximally to face stimuli (N170) [23-28]. An earlier P1 evoked at 100–120 ms was also reported to reflect face processing [25].

These face-evoked EEG and MEG responses with different response latencies imply the existence of different neural sub-processes underlying face perception. Because electric and magnetic fields recorded from the scalp surface or sensors near the scalp are summations of cortical activities (this statement is less true of MEG than it is of EEG), cortical responses evoked by a face stimulus should contain not only face-specific components [2,10], but also components related to basic visual features such as changes in luminance or non-specific responses such as those related to the detection of change accompanied by passive shifts of attention [29]. For instance, responses evoked by a stimulus are destined to be associated with processes such as an orienting response or passive attention because of the intrinsic property of the methodologies. In fact, classical studies of evoked responses have long discussed the relationship between evoked responses and specific theories derived from the orienting response theory [30,31]. Also, in many natural scenes, responses evoked by seeing a face would involve neural activity sensitive to luminance.

Previous face studies have compared responses to faces, other objects and scrambled faces, or manipulated a variety of factors affecting face recognition to examine face selectivity or other importance issues on face recognition [14,15,20,22,25,26,32]. In addition, a large number of studies have revealed the generators of face-related responses [16,21,28,32-34]. However, these paradigms cannot reveal which subcomponents whole-head activity for a face includes. For example, most previous studies examining face selectivity have also taken a subcomponent such as luminance-related activity into account by comparing cortical response to faces with other objects with the same luminance, but have not attempted to extract luminance-related sub-processes from the recorded activity. In this study, we attempted to segregate different components, luminance-related, face-related and non-specific, involved in the recorded activity in response to a face stimulus. To this end, we used whole-head MEG to record cortical responses evoked by each of three kinds of face stimuli; appearance of a face (Onset), disappearance of the face (Offset), and change from one face to another (Change) against a uniform background. The results of comparisons among these responses were hypothesized as follows. (1) Responses in brain areas involved in face recognition will not appear for Offset. (2) Responses in areas involved in changes in mean luminance will be smaller for Change than for the other two stimuli, because Change occurred without a change in mean luminance. (3) Finally, responses in areas involved in non-specific processes such as the detection of abrupt changes will appear commonly to all stimuli. The segregation of cortical responses related to basic visual, face-related and non-specific features from the recorded activity, would promote the understanding of face-related neural processing.

## Methods

### Subjects

Recordings were obtained from 14 healthy right-handed subjects (seven males, seven females), aged 25–55 years old (mean 35.4 ± 10.4). The present study was approved in advance by the Ethics Committee of the National Institute for Physiological Sciences, Okazaki, Japan, and written consent was obtained from all subjects.

### MEG recording

MEG was recorded with a helmet-shaped 306-channel detector array (Vectorview, Elekta Neuromag Yo, Helsinki, Finland), which consisted of 102 identical triple-sensor elements. Recordings were filtered with a band-pass filter of 0.1–200 Hz and digitized at a sampling rate of 1000 Hz. Before subjects entered the shielded room, three anatomical landmarks (nasion and bilateral preauricular points) were digitized using a 3-D digitizer. Then, four head-position-indicator (HPI) coils attached to the subject's head as well as several points (30–40 points) on the scalp were digitized with respect to the three anatomical landmarks. After digitization, the subject was seated in a magnetically shielded and darkened room. The subject's head was placed in the dewar and the shielded room door closed. The condition of all sensors was carefully checked and then a current was fed to four HPI coils and the resulting magnetic fields were measured with magnetometers to know the locations of the four HPI coils in the sensor coordinate system. The main experiment started after this procedure had been finished.

The period of analysis was from 100 ms before to 500 ms after the event. In our system, there are no trial-to-trial jitters, and the actual timing of the presentation of a visual stimulus on the projector is delayed 32 ms every trial in relation to a trigger signal from the computer. Accordingly, the MEG signal was shifted 32 ms every trial, and then averaged online. Trials with eye blinks monitored by an eye-movement monitor camera (ISCAN, Burlington, MA) and with MEG signals > 3000 fT/cm were automatically rejected. The signal space projection (SSP) technique was also used for removal of noises involved in the recorded signal.

### Stimuli

**Figure 1:**
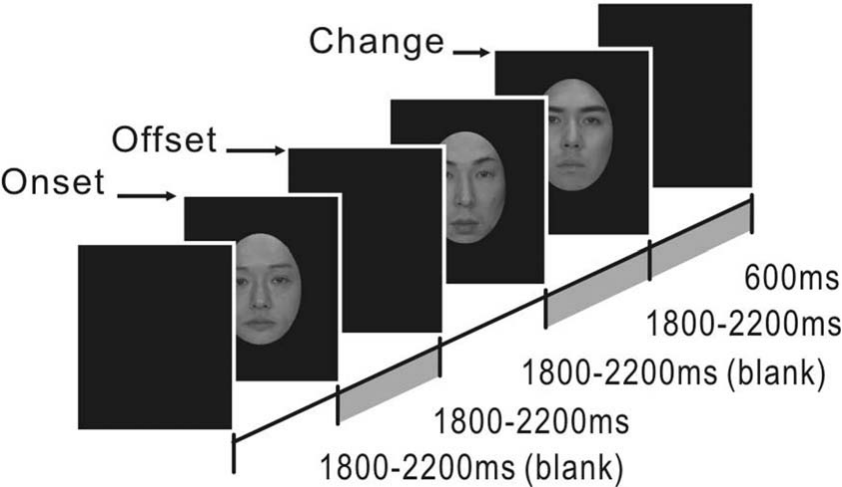
**The presentation sequence of face stimuli**.

The subjects, seated in a magnetically shielded and darkened room, were instructed to just watch a visual stimulus presented at the center of the screen in front of them. The stimulus was presented on the screen via a digital light processing projector placed outside the shielded room (Mirage 2000, CHRISTIE DIGITAL SYSTEM Inc., Kitcherner Canada). The refresh rate of the projector was 60 Hz. The viewing distance from subjects to the screen was 240 cm. Grayscale images of three persons were used as face stimuli. The pictures were presented within an oval window (vertical, 25 cm; horizontal, 18 cm) on a black background in two conditions (Fig. 1). In one condition, one of the pictures was presented for 1800–2200 ms. The appearance and disappearance of the face in this condition were referred to as "Onset" and "Offset", respectively. In the other condition, the presentation of one of the pictures for 1800–2200 ms was followed immediately by the presentation of another picture for 600 ms. The abrupt transition from one face to another in this condition was referred to as "Change". The interval from the offset of a condition to the onset of the next condition was 1800–2200 ms. The luminance of these faces was 4.2 cd/cm^2^. One hundred and twenty trials for each condition were randomly presented. A "rest" picture was presented for 5000 ms every 10 trials to reduce fatigue. To reduce the effects of cognitive factors, subjects were not required to perform any tasks.

### Data analysis

First, we calculated vector sums from the longitudinal and latitudinal derivatives of magnetic fields passing through each of the planar gradiometers. This was achieved by squaring MEG signals for each of two gradiometers at a sensor's location, summing the squared signals, and then calculating the root of the sum, that is:



This is here called the root sum square, RSS [35] and corresponds to a scalar product (inner product) of two vectors. This calculation was carried out for all 102 sensor locations. RSS waveforms at all 102 sensor locations were carefully examined to find distinct source activities with different temporal and spatial properties. The RSS signal best reflects the strength of magnetic fields just below a sensor's location, and the peak of its spatial distribution shows the location nearest the source of activation because of the properties of planar gradiometers. After examination of the RSS waveform and the field distribution pattern at some RSS peaks, source locations and the time course of source activities were determined by a multiple source analysis method, brain electric source analysis (BESA, MEGIS Software GmbH, German), as described previously [36-38]. Model adequacy was assessed by examining 1) F-ratio (ratio of reduced chi-square values before and after adding a new source) [39], 2) residual waveforms (difference between the recorded data and the model), and 3) RSS waveform and topography. The integral probability of obtaining an F-ratio value equal to or greater than the obtained value is calculated to evaluate whether a model with a larger number of dipoles represents a statistically significant improvement of the fit over a model with a smaller number of dipoles. When a P value was smaller than 0.05, we considered the new dipole as significant. We continued to add a source to the model until the addition of a dipole did not significantly improve the fit. The procedure to assess model accuracy was basically the same as described elsewhere [36,40]. Estimated dipoles were projected onto individual MR images constructed by Brain Voyager (QX 1.4, Maastricht, the Netherlands). The locations of the dipoles were transformed to Talairach coordinates by coregistration of BESA and Brain Voyager.

To compare the difference in peak latency or amplitude of each source activity, analysis of variance (ANOVA) was performed. The level of statistical significance was set at P < 0.05. When the sphericity assumption was violated, Greenhouse-Geisser correction coefficient epsilon was used to correct the degrees of freedom, and then the F-value and significance probability were re-calculated.

## Results

**Figure 2:**
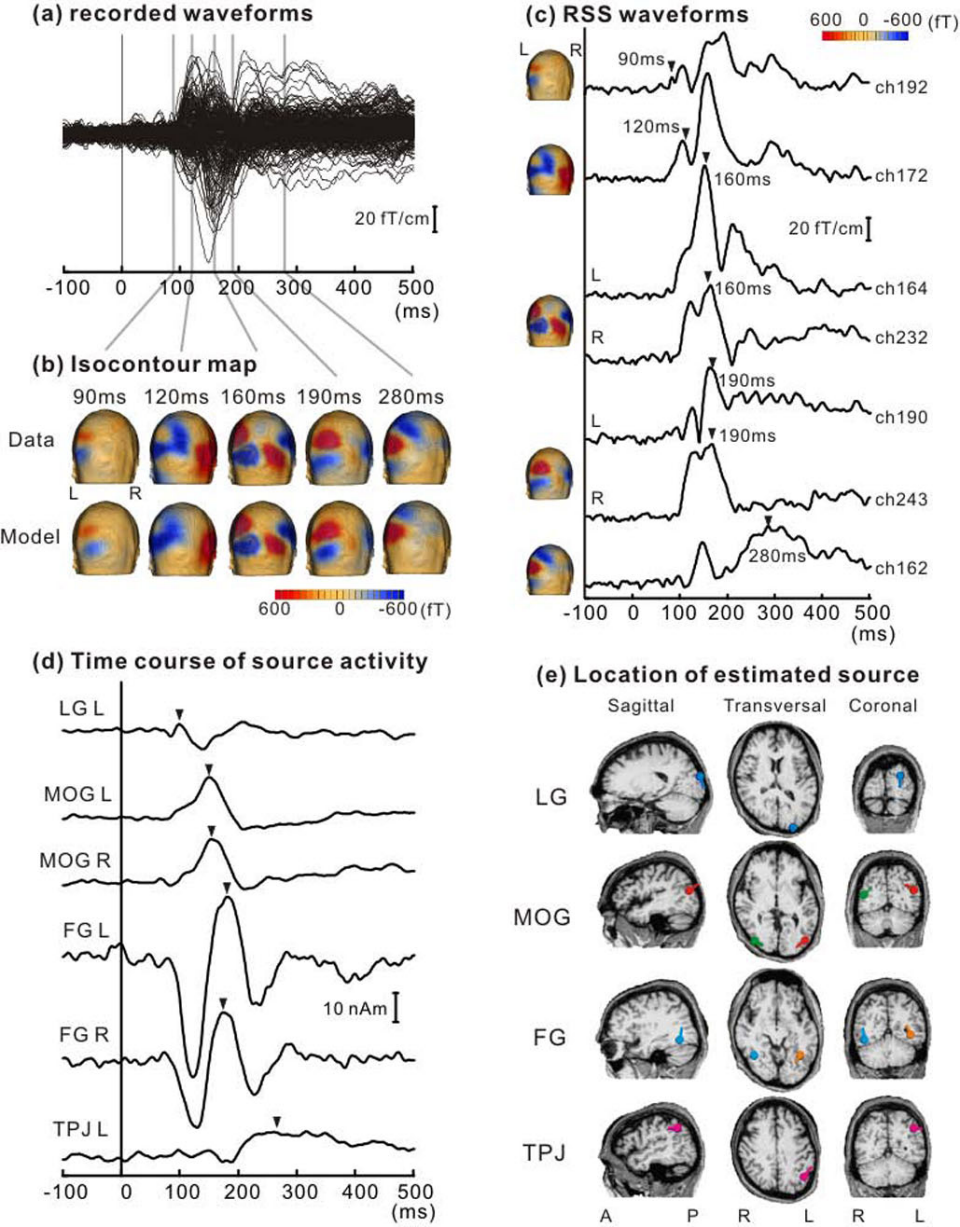
**Analysis in the present study of a representative subject**. Data for Onset are shown. (A) Superimposed waveforms recorded from 204 planar gradiometers. (B) Isocontour maps of recorded data (Data) and the model (Model) drawn on the subject's head at 5 latency points. The two isocontour maps well fit each other. (C) Waveforms of root sum square (RSS). (D) Source strength as a function of time. (E) The locations of estimated dipole sources superimposed on the subject's MR image. Bars of dipoles indicate the direction of upward deflections. LG, lingual gyrus; MOG, middle occipital gyrus; FG, fusiform gyrus; TPJ, temporo-parietal junction; L, Left hemisphere; R, Right hemisphere.

In all subjects, clear MEG responses were recorded for Onset, Offset, and Change. RSS waveforms of each subject showed several peaks at different sensor locations and different latencies, suggesting the presence of at least several distinct source activities. Figure 2 shows the original waveforms, RSS waveforms and isocontour maps at several RSS peaks obtained in a representative subject in response to Onset. First, analysis procedures are explained using data from this subject, and then the results from all subjects will be presented.

### Analysis procedures

The largest response to Onset was observed at around 160 ms (M160) in occipito-temporal regions, whose magnetic field distribution generally showed a symmetric two-dipole pattern. M160 was preceded by a smaller response at 120 ms (M120) with a different dipole pattern from M160. A later response with an opposite polarity to that for M160 was observed at around 200–300 ms (M250) in a slightly superior and anterior region to M160. These magnetic field distributions suggested that at least three distinct sources exist in each hemisphere.

To differentiate overlapping cortical activities, waveforms were analyzed by a multiple source method. Dipoles were fitted one by one around the peak of these individual responses with the aid of the RSS waveform and the topography. Figure 2D shows the time course of each cortical activity. Figure 2E shows the location and orientation of each source superimposed on the subject's magnetic resonance (MR) images. The source responsible for M120 was located in the bilateral FG. Waveforms of FG activity showed a triphasic pattern peaking at around 120 ms, 190 ms and 250 ms. The source responsible for M160 was located in the bilateral MOG. The source responsible for M250 was located around the left temporo-parietal junction (TPJ). By applying our criteria, an additional source could be included in the model to explain the residual waveforms, which was located in the left lingual gyrus (LG). Accordingly, we successfully estimated activity in the LG, MOG, FG, and TPJ for Onset. Figure 2B depicts isocontour maps at several latency points of the recorded data and the model.

### Comparison among responses to different stimuli

**Figure 3:**
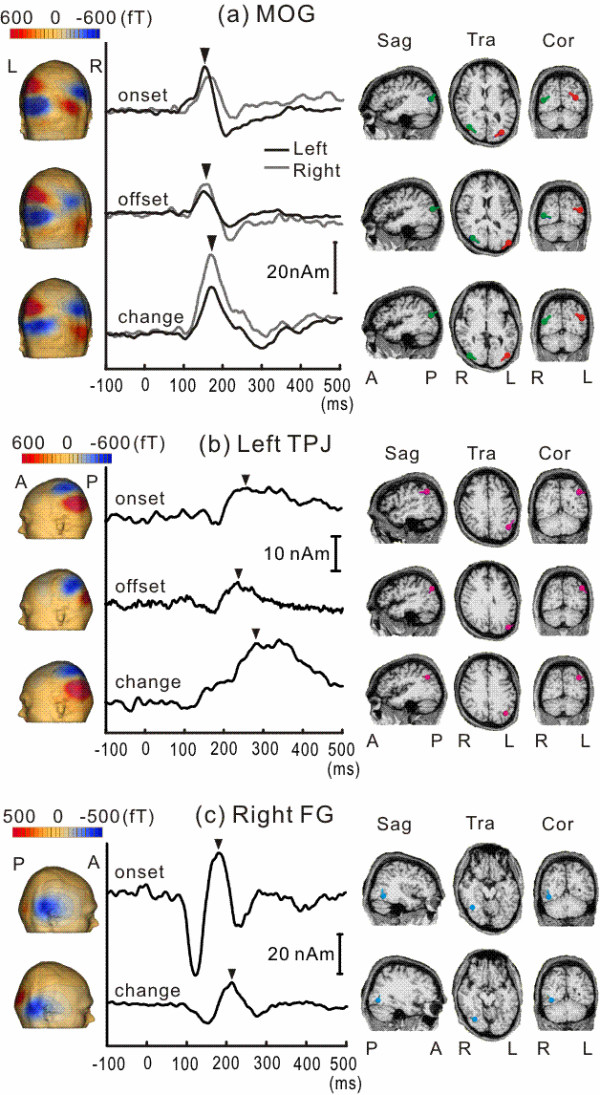
**Comparison of the isocontour map, time course of source activity, and locations of dipoles among Onset, Offset and Change for each cortical activity**. Note the very similar activation profiles of MOG and TPJ activity among the three conditions.

Similar procedures were applied to the responses to the other two stimuli. Figure 3 shows the locations of sources, the time course of source activity, and the isocontour map of each cortical activity for Onset, Offset, and Change. The three stimuli all evoked very similar activity in MOG with respect to the location, time course of source activity, and isocontour map (Figure 3A). TPJ activity was also evoked by each of the stimuli, and had a similar profile among them (3B). FG activity was evoked by Onset and Change but not by Offset (3C).

### Results from all subjects

**Figure 4:**
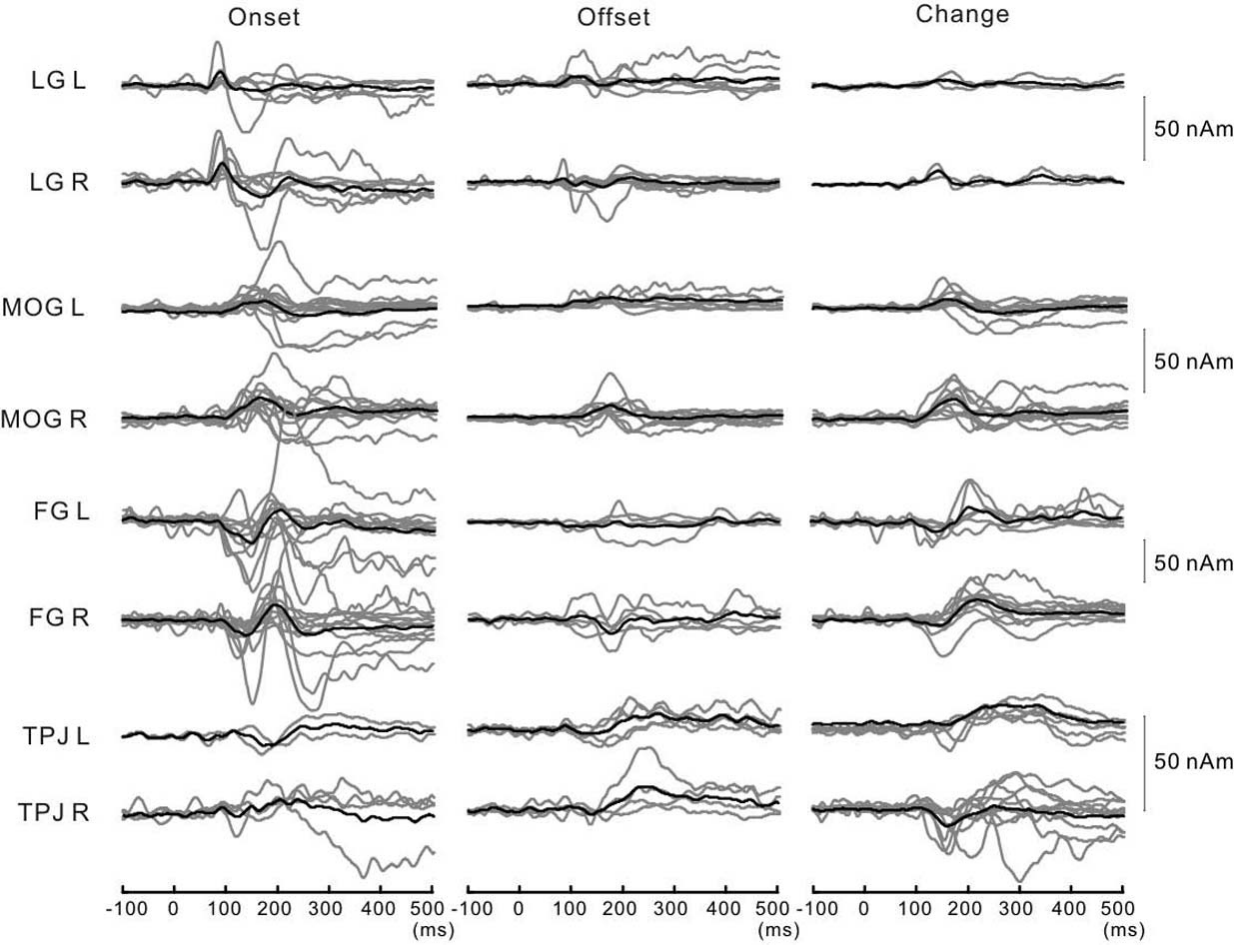
**The time course of each source activity for all subjects**. Gray and black lines indicate waveforms of each subject and their grand average, respectively.

Similar procedures were applied to the data from the remaining subjects. By applying our criteria, two to seven sources were included in the model for each subject. The estimated dipoles for each subject were classified based on their locations and time courses of activities (Figure 4). The mean Talairach coordinates across subjects are shown in Table 1. Other sources, such as that in the cingulate or inferior frontal cortex, were found only in a limited number of subjects, and were not included in the analyses. Table 2 shows the mean peak latency of each activity. The source of activity in the LG was identified in about half of the subjects for Onset and Offset, but was very rare for Change. The source in the MOG was identified for all stimuli in many subjects. The source of FG was identified for Onset and Change but in fewer subjects than the source in the MOG, and was not identified for Offset. The source of TPJ activity was identified for all three stimuli but in more subjects for Change than the other stimuli.

**Table 1:** Talairach coordinates of each source.

	Onset			Offset			Change		
Source	x	y	Z	x	y	z	x	y	z

LG L	-13 (4)	-88 (2)	-3 (5)	-13 (4)	-84 (3)	3 (4)	-	-	-
LG R	10 (5)	-78 (5)	-10 (4)	12 (4)	-89 (4)	-3 (4)	7 (-)	-95 (-)	-12 (-)
MOG L	-34 (4)	-73 (4)	3 (5)	-38 (5)	-74 (6)	8 (7)	-43 (2)	-70 (5)	2 (4)
MOG R	29 (3)	-70 (4)	2 (4)	35 (4)	-66 (4)	1 (4)	36 (3)	-62 (5)	4 (3)
FG L	-30 (3)	-53 (3)	-13 (3)	-	-	-	-34 (3)	-52 (6)	-14 (1)
FG R	30 (3)	-55 (4)	-13 (3)	-	-	-	31 (1)	-54 (1)	-13 (1)
TPJ L	-47 (6)	-45 (13)	26 (11)	-48 (4)	-53 (3)	7 (3)	-46 (4)	-46 (7)	17 (5)
TPJ R	42 (2)	-38 (9)	24 (17)	42 (4)	-43 (11)	19 (10)	49 (2)	-40 (5)	17 (5)

Values are expressed as the means (SE). LG, lingual gyrus; MOG, middle occipital gyrus; FG, fusiform gyrus; TPJ, temporo-parietal junction.

**Table 2:** Peak latency of each source activity.

	Latency (ms)			N		
Source	Onset	Offset	Change	Onset	Offset	Change

LG L	87 (3)	104 (6)	-	7	5	0
LG R	93 (4)	119 (18)	95 (-)	5	7	1
MOG L	151 (7)	152 (13)	152 (6)	13	7	9
MOG R	149 (7)	148 (6)	162 (5)	11	10	11
FG L	182 (10)	-	200 (7)	11	0	7
FG R	175 (10)	-	211 (8)	12	0	10
TPJ L	277 (35)	268 (15)	250 (11)	2	5	6
TPJ R	226 (4)	222 (5)	243 (11)	4	4	10

The number of subjects whose source was successfully estimated in each cortical area is also shown (N). Values are expressed as the means (SE).

For two-way analysis of variance (ANOVA, laterality * stimulus, see Methods) of the peak latency of FG activity, there was a significant main effect of the stimulus [F (1, 32) = 10.7, P < 0.01], such that the peak latency for Change (left, 200 ms; right, 211 ms) was significantly increased compared to that for Onset (left, 182 ms; right, 175 ms). Results of ANOVA showed no significant differences in latency for other source activities. For the dipole moment of each source obtained in the source analysis, two-way ANOVA showed no significant differences.

## Discussion

The present study recorded cortical responses to face appearance (Onset), disappearance (Offset), and change (Change) using MEG. We found activity in the MOG and TPJ for each of the three stimuli, and in the LG for Onset and Offset but not for Change. Activity in the FG was evoked by Onset and Change but not by Offset.

### Middle occipital gyrus (MOG)

Activity in the MOG at 150 ms was the stable main activity found for all stimuli, possibly corresponding to V3. Because Offset should not activate face recognition processes that are triggered by the appearance of a face, the present results of activity in the MOG for both Onset and Offset suggest that it is cortical activity non-specific to face processing. Moreover, the stable activation in response to Change without a change in mean luminance suggests that MOG activity is associated with general visual function such as change detection in the visual system irrespective of changes in mean luminance. These results are consistent with previous findings that MOG was activated by any visual stimulus [29]. Our recent study also found a similar time course of activation in the MOG in response to a simple visual stimulus (star) [41], consistent with the notion that MOG activity is related to general visual function. Our present and previous data showed that MOG activity was not generated specifically by the appearance or change of a face.

Because we did not compare faces and other objects, we cannot comment on face selectivity, but the latency of the MOG response seems to be very similar to the ERP N170 component. Most previous studies have reported that the peak latency of N170 in response to an upright, neutral, and unmasked face stimulus was actually earlier than 170 ms, e.g., 156 ms [25], 160 ms [42], 161 ms [43], 162 ms [34], 164 ms [44], and 158 ms [32]. A recent study investigated ERP responses to changes of face identity using the alternative presentation of different faces [45]. This ERP study reported that N170 peaking at about 160 ms was greater when the preceding face stimulus was perceived as having a different identity than when perceived as having the same identity. The present study revealed one of the cortical sources of MEG responses to a face change as well as onset and offset of a face. Future studies to compare different stimulus appearances between face and non-face objects using the procedure in the present study are valuable to seek out neural processes of face recognition in detail.

### Temporoparietal junction (TPJ)

Activity in the TPJ was evoked with a relatively long duration, peaking at around 250 ms by all three stimuli. As in the MOG, the common activity in the TPJ for Onset, Offset and Change suggests that it is associated with non-specific processing, like the detection of change in the visual system irrespective of changes in mean luminance. The TPJ, including the superior temporal gyrus (STG) and supra-marginal gyrus (SMG), responds to changes or salience of sensory stimuli in any sensory modality [29,46,47]. fMRI studies have emphasized the importance of the TPJ in multimodal integration [48,49]. On the other hand, the MOG is considered a unimodally responsive visual area [29,46,47]. The TPJ would reflect higher-order processing than the MOG. The longer response latency of the TPJ than MOG shows that TPJ activity is associated with higher-order processing than MOG, although both the TPJ and MOG responded to all three stimuli.

### Fusiform gyrus (FG)

The triphasic waveform of the FG in the present study was consistent with the results of intracranial ERP studies by Allison and colleagues [10-12]. They found a triphasic response (P150-N200-P290) in the FG to face onset stimuli. Our measured triphasic activity is consistent with their recorded intracranial potentials, with respect to the latency, time course, and polarity. Their intracranial recordings have also demonstrated even larger responses in the FG to face stimuli than other visual stimuli. Consistent with this, some neurons in the inferotemporal (IT) area have been demonstrated to respond selectively to the presentation of a face or face parts, or simultaneous presentation of multiple parts [50,51]. Thus, Allison et al. (2002) [52] assumed that there are hypothetical face-selective neurons in a restricted part of the human FG which respond selectively to faces but not to words, and that N200 is caused by depolarization of apical dendrites of these neurons in layers 3 and 4. fMRI studies have also observed face-selective activations in FG [2,4-8], and some authors have called this face-selective area in the FG, the fusiform face area (FFA) [4]. In the present study, we found triphasic FG activity in response to Onset and Change, while Offset did not activate FG. By contrast, MOG and TPJ activities were found for all three stimuli. In addition, our EEG study demonstrated similar triphasic responses of the FG to a simple star-shaped visual stimulus [41]. In general, IT area, including FG, is considered to be involved in the recognition of non-face objects as well as faces [53,54]. These results suggest that, unlike activities in the MOG, TPJ and LG (described below), the FG activity observed in this study constitutes some of the neural processes selectively initiated by the appearance of a face or different face or objects, which in turn may not be associated with processes when the face disappears (e.g., the peek-a-boo).

### Lingual gyrus (LG)

Little if any activity of the LG was found for Change, consistent with our prediction, which suggests that this activity would be related to changes in mean luminance. The location and orientation of the estimated dipoles might indicate that the activity reflects the activity of V1/V2 [37]. One may consider that it was not possible to locate V1/V2 in most subjects because the stimuli were large and centrally presented. In this case, the simultaneous activation from neurons in the upper and lower banks of the calcarine sulcus might be cancelled out. However, this would not explain why Change alone did not evoke the V1/V2 activity, because the stimuli were all presented centrally and of the same size. An intracranial recording study also revealed a prominent response from electrodes implanted in the peri-calcarine sulcus in response to both onset and offset stimuli [55], thereby supporting the present results for Onset and Offset.

### Methodological limitation

In the present study, we aimed to compare MEG responses among Onset, Offset and Change. As Onset has to precede Offset, randomization of the order of the 3 stimuli is incomplete, and therefore we randomized the order of 2 sequences including the 3 stimuli (one for Onset and Offset, another for Change). However, the pre-offset duration was long enough to clearly evoke the offset response. Another methodological issue is that we used just three pictures. Effects of repetition have been reported in previous studies using a paired stimulus paradigm, with decreased responses to repeated stimulation. However, our stimuli were presented with a relatively long interval (about 2 sec). Therefore, we are not sure whether repetition had similar effects in our study. Also, to minimize long-term habituation which reduces neural activity as an experiment proceeds, we measured the responses as quickly as possible (in less than 30 min). Still, we cannot completely rule out the possibility that some activity was reduced with stimulus repetition as the experiment continued. Also, all images used in this study were of males to exclude gender differences, and in turn there might be some results specific to males.

## Conclusion

The present study suggested that activity in the MOG is related to a more general function in the visual system, such as the detection of stimulus changes. We found triphasic activation in the FG in response to Onset and Change, which corresponds to intracranial potential in the FG in previous intracranial recordings. These results show that activity of the FG is related to object recognition as an important module in the visual ventral stream. Activity of the LG was related to changes in luminance. Long-lasting activity of the TPJ is related to neural processes underlying the detection of stimulus changes at the higher-order stage. In summary, the present study revealed four different neural processes involved in activity evoked by a face stimulus (luminance change, object recognition, and stimulus change). The combination of the analysis and stimulus used in this study also assures the usefulness of MEG when investigating the human sensory system.

## Abbreviations

BESA: brain electric source analysis; EEG: electroencephalography; ERP: event-related potential; FFA: fusiform face area; FG: fusiform gyrus; fMRI: functional magnetic resonance imaging; HPI: head position indicator; IT: inferotemporal; LG: Lingual Gyrus; MEG: magnetoencephalography; MOG: middle occipital gyrus; PET: positron emission tomography; RSS: root sum square; SMG: supra-marginal gyrus; STG: superior-temporal gyrus; TPJ: temporo-parietal junction.

## Authors' contributions

ET contributed to data collection, data analysis, and drafting the paper. KI contributed to planning the study, and revising the paper. TK and RK contributed to revising the paper. All authors read and approved the final manuscript.

## References

[B1] Sergent J, Ohta S, MacDonald B (1992). Functional neuroanatomy of face and object processing. A positron emission tomography study. Brain.

[B2] Puce A, Allison T, Gore JC, McCarthy G (1995). Face-sensitive regions in human extrastriate cortex studied by functional MRI. Journal of neurophysiology.

[B3] Haxby JV, Ungerleider LG, Horwitz B, Maisog JM, Rapoport SI, Grady CL (1996). Face encoding and recognition in the human brain. Proceedings of the National Academy of Sciences of the United States of America.

[B4] Kanwisher N, McDermott J, Chun MM (1997). The fusiform face area: a module in human extrastriate cortex specialized for face perception. J Neurosci.

[B5] Rotshtein P, Henson RN, Treves A, Driver J, Dolan RJ (2005). Morphing Marilyn into Maggie dissociates physical and identity face representations in the brain. Nature neuroscience.

[B6] Loffler G, Yourganov G, Wilkinson F, Wilson HR (2005). fMRI evidence for the neural representation of faces. Nature neuroscience.

[B7] George N, Dolan RJ, Fink GR, Baylis GC, Russell C, Driver J (1999). Contrast polarity and face recognition in the human fusiform gyrus. Nature neuroscience.

[B8] Halgren E, Dale AM, Sereno MI, Tootell RB, Marinkovic K, Rosen BR (1999). Location of human face-selective cortex with respect to retinotopic areas. Human brain mapping.

[B9] Allison T, Ginter H, McCarthy G, Nobre AC, Puce A, Luby M, Spencer DD (1994). Face recognition in human extrastriate cortex. Journal of neurophysiology.

[B10] Allison T, Puce A, Spencer DD, McCarthy G (1999). Electrophysiological studies of human face perception. I: Potentials generated in occipitotemporal cortex by face and non-face stimuli. Cereb Cortex.

[B11] McCarthy G, Puce A, Belger A, Allison T (1999). Electrophysiological studies of human face perception. II: Response properties of face-specific potentials generated in occipitotemporal cortex. Cereb Cortex.

[B12] Puce A, Allison T, McCarthy G (1999). Electrophysiological studies of human face perception. III: Effects of top-down processing on face-specific potentials. Cereb Cortex.

[B13] Allison T, McCarthy G, Nobre A, Puce A, Belger A (1994). Human extrastriate visual cortex and the perception of faces, words, numbers, and colors. Cereb Cortex.

[B14] Liu J, Harris A, Kanwisher N (2002). Stages of processing in face perception: an MEG study. Nat Neurosci.

[B15] Itier RJ, Herdman AT, George N, Cheyne D, Taylor MJ (2006). Inversion and contrast-reversal effects on face processing assessed by MEG. Brain research.

[B16] Linkenkaer-Hansen K, Palva JM, Sams M, Hietanen JK, Aronen HJ, Ilmoniemi RJ (1998). Face-selective processing in human extrastriate cortex around 120 ms after stimulus onset revealed by magneto- and electroencephalography. Neuroscience letters.

[B17] Watanabe S, Kakigi R, Puce A (2003). The spatiotemporal dynamics of the face inversion effect: a magneto- and electro-encephalographic study. Neuroscience.

[B18] Watanabe S, Kakigi R, Koyama S, Kirino E (1999). Human face perception traced by magneto- and electro-encephalography. Brain Res Cogn Brain Res.

[B19] Miki K, Watanabe S, Kakigi R, Puce A (2004). Magnetoencephalographic study of occipitotemporal activity elicited by viewing mouth movements. Clin Neurophysiol.

[B20] Liu J, Higuchi M, Marantz A, Kanwisher N (2000). The selectivity of the occipitotemporal M170 for faces. Neuroreport.

[B21] Halgren E, Raij T, Marinkovic K, Jousmaki V, Hari R (2000). Cognitive response profile of the human fusiform face area as determined by MEG. Cereb Cortex.

[B22] Furey ML, Tanskanen T, Beauchamp MS, Avikainen S, Uutela K, Hari R, Haxby JV (2006). Dissociation of face-selective cortical responses by attention. Proceedings of the National Academy of Sciences of the United States of America.

[B23] Eimer M (2000). Effects of face inversion on the structural encoding and recognition of faces. Evidence from event-related brain potentials. Brain Res Cogn Brain Res.

[B24] Eimer M (2000). Event-related brain potentials distinguish processing stages involved in face perception and recognition. Clin Neurophysiol.

[B25] Itier RJ, Taylor MJ (2004). N170 or N1? Spatiotemporal differences between object and face processing using ERPs. Cereb Cortex.

[B26] Bentin S, Allison T, Puce A, Perez E, McCarthy G (1996). Electrophysiological studies of face perception in humans. Journal of Cognitive Neuroscience.

[B27] Bentin S, Deouell LY, Soroker N (1999). Selective visual streaming in face recognition: evidence from developmental prosopagnosia. Neuroreport.

[B28] Bötzel K, Schulze S, Stodieck SR (1995). Scalp topography and analysis of intracranial sources of face-evoked potentials. Exp Brain Res.

[B29] Downar J, Crawley AP, Mikulis DJ, Davis KD (2000). A multimodal cortical network for the detection of changes in the sensory environment. Nature neuroscience.

[B30] Naatanen R, Picton T (1987). The N1 wave of the human electric and magnetic response to sound: a review and an analysis of the component structure. Psychophysiology.

[B31] Donchin E, Coles MG (1988). Is the P300 component a manifestation of context updating?. Brain Behav Sci.

[B32] Deffke I, Sander T, Heidenreich J, Sommer W, Curio G, Trahms L, Lueschow A (2007). MEG/EEG sources of the 170-ms response to faces are co-localized in the fusiform gyrus. Neuroimage.

[B33] Tanskanen T, Nasanen R, Montez T, Paallysaho J, Hari R (2005). Face recognition and cortical responses show similar sensitivity to noise spatial frequency. Cereb Cortex.

[B34] Rossion B, Joyce CA, Cottrell GW, Tarr MJ (2003). Early lateralization and orientation tuning for face, word, and object processing in the visual cortex. NeuroImage.

[B35] Kida T, Inui K, Wasaka T, Akatsuka K, Tanaka E, Kakigi R (2007). Time-varying cortical activations related to visual-tactile cross-modal links in spatial selective attention. Journal of neurophysiology.

[B36] Inui K, Wang X, Tamura Y, Kaneoke Y, Kakigi R (2004). Serial processing in the human somatosensory system. Cereb Cortex.

[B37] Inui K, Kakigi R (2006). Temporal analysis of the flow from V1 to the extrastriate cortex in humans. Journal of neurophysiology.

[B38] Scherg M, Buchner H (1993). Somatosensory evoked potentials and magnetic fields: separation of multiple source activities. Physiological measurement.

[B39] Supek S, Aine CJ (1993). Simulation studies of multiple dipole neuromagnetic source localization: model order and limits of source resolution. IEEE Trans Biomed Eng.

[B40] Inui K, Okamoto H, Miki K, Gunji A, Kakigi R (2006). Serial and parallel processing in the human auditory cortex: a magnetoencephalographic study. Cereb Cortex.

[B41] Tanaka E, Inui K, Kida T, Miyazaki T, Takeshima Y, Kakigi R (2008). A transition from unimodal to multimodal activations in four sensory modalities in humans: an electrophysiological study. BMC neuroscience.

[B42] Jacques C, Rossion B (2007). Early electrophysiological responses to multiple face orientations correlate with individual discrimination performance in humans. NeuroImage.

[B43] Latinus M, Taylor MJ (2006). Face processing stages: impact of difficulty and the separation of effects. Brain research.

[B44] Anaki D, Zion-Golumbic E, Bentin S (2007). Electrophysiological neural mechanisms for detection, configural analysis and recognition of faces. NeuroImage.

[B45] Jacques C, Rossion B (2006). The speed of individual face categorization. Psychol Sci.

[B46] Downar J, Crawley AP, Mikulis DJ, Davis KD (2002). A cortical network sensitive to stimulus salience in a neutral behavioral context across multiple sensory modalities. Journal of neurophysiology.

[B47] Downar J, Crawley AP, Mikulis DJ, Davis KD (2001). The effect of task relevance on the cortical response to changes in visual and auditory stimuli: an event-related fMRI study. NeuroImage.

[B48] Beauchamp MS, Argall BD, Bodurka J, Duyn JH, Martin A (2004). Unraveling multisensory integration: patchy organization within human STS multisensory cortex. Nature neuroscience.

[B49] Calvert GA (2001). Crossmodal processing in the human brain: insights from functional neuroimaging studies. Cereb Cortex.

[B50] Baylis GC, Rolls ET, Leonard CM (1987). Functional subdivisions of the temporal lobe neocortex. J Neurosci.

[B51] Perrett DI, Rolls ET, Caan W (1982). Visual neurones responsive to faces in the monkey temporal cortex. Experimental brain research Experimentelle Hirnforschung.

[B52] Allison T, Puce A, McCarthy G (2002). Category-sensitive excitatory and inhibitory processes in human extrastriate cortex. Journal of neurophysiology.

[B53] Tanaka K (1996). Inferotemporal cortex and object vision. Annual review of neuroscience.

[B54] Logothetis NK, Sheinberg DL (1996). Visual object recognition. Annual review of neuroscience.

[B55] Huettel SA, McKeown MJ, Song AW, Hart S, Spencer DD, Allison T, McCarthy G (2004). Linking hemodynamic and electrophysiological measures of brain activity: evidence from functional MRI and intracranial field potentials. Cereb Cortex.

